# The Effectiveness of Multi-Component Support Programs for Alzheimer’s Caregivers on Burden: A Systematic Review and Meta-Analysis

**DOI:** 10.1192/j.eurpsy.2025.883

**Published:** 2025-08-26

**Authors:** B. Şentürk Kiliç, D. Hiçdurmaz, S. Karahan

**Affiliations:** 1 Faculty of Nursing; 2 Faculty of Medicine/Biostatistics, Hacettepe University, Ankara, Türkiye

## Abstract

**Introduction:**

Alzheimer’s disease, necessitates continuous, long-term care primarily provided by family members. As the disease progresses, caregivers experience increasing stress and burden. Research indicates that caregivers of Alzheimer’s patients are at higher risk for depression, social isolation, and health problems. In order to help the caregivers, multi-component support programs, which integrate approaches such as education, psychoeducation, and social support, have shown more positive outcomes. However, systematic reviews and meta-analyses examining the impact of these programs remain limited.

**Objectives:**

This systematic review and meta-analysis aimed to evaluate the effectiveness of multi-component support programs on the caregiver burden of individuals caring for Alzheimer’s patients.

**Methods:**

The research was conducted through searches in five databases (CENTRAL, CINAHL, PsycINFO, PubMed, WOS), focusing on randomized controlled trials that met the inclusion criteria. Two researchers independently evaluated the full texts, assessing risk of bias with the Cochrane ‘Risk of Bias-2’ tool and evidence quality using the GRADE tool. Participants included individuals aged 18 and older who were the primary caregivers for those diagnosed with Alzheimer’s disease and had provided care for at least three months. The intervention included at least two types of support, such as skill training, education, counseling, or therapy. The primary outcome was caregiver burden.

**Results:**

The review included 8 studies overall. Among the 1147 participants, only one study was web-based, while the other seven interventions were conducted face-to-face. The components of the interventions were mainly educational, supportive, and skill-building, with only one intervention including respite care. Overall risk of bias assessment recorded one study with high risk, four with unclear risk, and one with low risk. The effect sizes of the interventions were calculated based on the means and standard deviations of caregiver burden scores before and after the intervention, as well as follow-up measurements. The multi-component intervention programs were found to have an uncertain short-term effect (Cohen’s d = 0.12; 95% CI: -0.06 - 0.29; p = 0.39) but were effective in the long term (Cohen’s d = 0.21; 95% CI: 0.03 - 0.38; p = 0.02). The certainty of evidence for caregiver burden outcomes was determined to be low before the intervention and follow-up, and very low from pre-intervention to post-intervention measurements. The data is current as of 12/12/2023.

**Image 1:**

**Figure:**
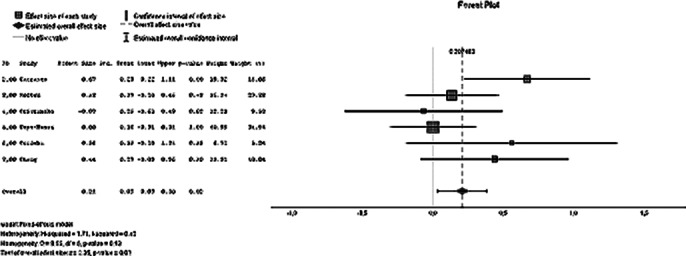

**Conclusions:**

Multi-component support programs are effective in reducing caregiver burden for Alzheimer’s caregivers in the long term; however, more high-quality studies are needed to confirm this effectiveness.

**Disclosure of Interest:**

None Declared

